# Acute Cardiogenic Shock Induced by Infusional 5-Fluorouracil

**DOI:** 10.1155/2014/819396

**Published:** 2014-10-28

**Authors:** Jeffrey Sulpher, Franco Dattilo, Susan Dent, Michele Turek, M. Neil Reaume, Christopher Johnson

**Affiliations:** ^1^Division of Medical Oncology, The Ottawa Hospital, Ottawa, ON, Canada K1H 8L6; ^2^Division of Cardiology, The Ottawa Hospital, Ottawa, ON, Canada K1H 8L6

## Abstract

A 49-year-old patient with metastatic carcinoma of the bladder and no prior history of heart disease presented with diffuse ST elevation, elevated troponins, and biventricular dysfunction requiring intensive care unit admission and inotropic support after receiving her first course of infusional 5-fluorouracil (5-FU). Over the course of several days, the patient's cardiac function and clinical status returned to baseline. A follow-up echocardiogram performed 5 days after initial presentation revealed an ejection fraction of 59 percent, with no evidence of wall motion abnormalities. Subsequent 5-FU chemotherapy was discontinued, and the patient went on to receive second-line chemotherapy.

## 1. Introduction

5-Fluorouracil (5-FU) chemotherapy can induce coronary artery vasospasm, even in patients without underlying ischemic heart disease. This syndrome can present with chest pain, ST elevation on electrocardiogram, and elevated troponins. Here we report a case of chemotherapy-induced acute cardiogenic shock, a rare and dramatic presentation of 5-FU related cardiac toxicity resulting in hypotension and multiorgan system dysfunction.

## 2. Case Report

A 49-year-old female with metastatic urothelial carcinoma of the bladder was transferred to our tertiary care institution from another hospital with hypoxia, tachycardia, and chest pain. Three days previously, she received her first cycle of palliative chemotherapy consisting of cisplatin (75 mg/m^2^) and infusional 5-fluorouracil (1000 mg/m^2^/day × 4 days). Her body surface area was 1.84 m^2^ and her body mass index was 26.9 kg/m^2^. She had no previous history of cardiac disease, diabetes, dyslipidemia, or smoking. Prior to transfer, the patient was assessed in the emergency department for nausea, vomiting, and hypovolemia. An initial working diagnosis of undifferentiated shock was made, for which she received four litres of intravenous crystalloid with minimal urine output.

**Figure 1:**
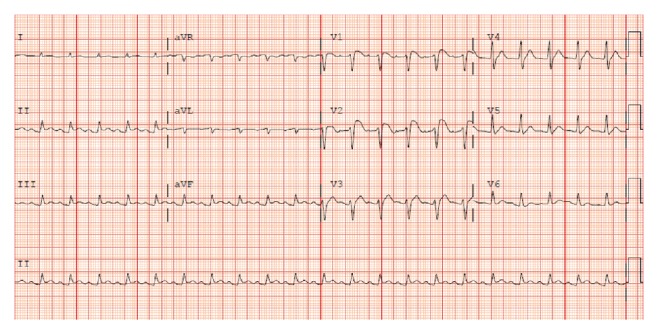
ECG May 20, 00:26.

Upon transfer, the patient was in respiratory distress, with an oxygen saturation of 93 percent measured by pulse oximetry, on 100 percent oxygen via nonrebreather facemask. She subsequently became cyanotic and hypotensive, requiring endotracheal intubation and mechanical ventilation. Blood pressure decreased to 96/52 mm Hg with clinical evidence of hypoperfusion and multiorgan system dysfunction, requiring vasopressor and inotropic support. A venous blood gas revealed a pH of 7.10, a pCO_2_ of 61 mm Hg, and an HCO_3_ of 19 mmol/L. Serum lactate and creatinine were elevated at 5.7 mmol/L and 361 *μ*mol/L, respectively. Serum troponin I was also elevated, with a peak value of 2.91 *μ*g/L 24 hours after transfer. Chest radiographs revealed bilateral areas of patchy consolidation with pleural effusions. A computed tomography pulmonary angiogram ruled out an embolism but confirmed bilateral airspace consolidation in all 5 lobes. Bronchoscopy and lavage were performed, which ruled out evidence of obstruction or infection. An electrocardiogram demonstrated ST segment elevation in the precordial leads and in the limb leads, with no reciprocal ST segment depression (Figure 1).

A transthoracic echocardiogram (while on inotropic support) revealed moderate biventricular dysfunction with a left ventricular ejection fraction of 44%. A provisional diagnosis of cardiogenic shock was established, and the patient was transferred to the intensive care unit for supportive care.

**Figure 2:**
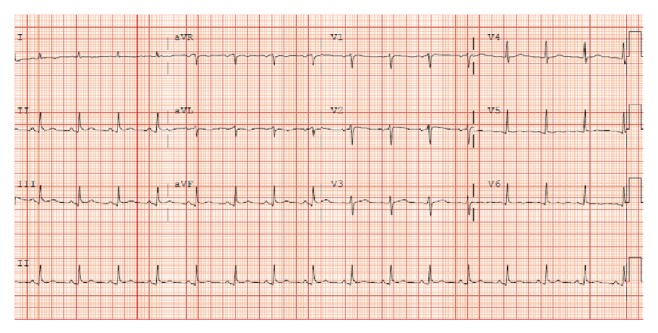
ECG May 20, 08:38.

**Figure 3:**
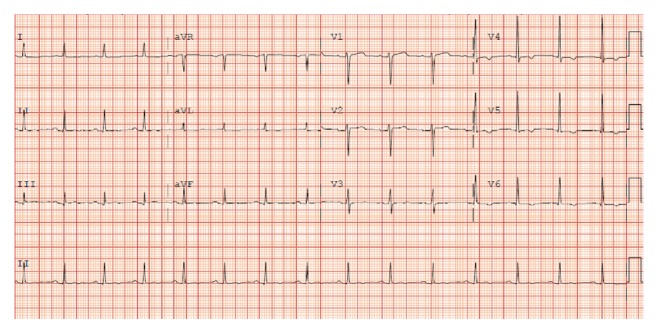
ECG May 24, 09:06.

While in the intensive care unit, intravenous milrinone, phenylephrine, norepinephrine, and vasopressin were required to maintain adequate blood pressure and cardiac output. Blood, urine, and sputum cultures were obtained, and empiric antibiotics were administered. The patient's renal function continued to deteriorate, and continuous renal replacement therapy was subsequently initiated. Over the course of several days, the patient's cardiac output and hypoxemic respiratory failure improved, vasopressors and inotropes were discontinued, and she was extubated and transitioned to intermittent hemodialysis. Her renal function slowly recovered, and hemodialysis was eventually discontinued. The initial ST segment changes resolved, with no evidence of Q waves on serial ECGs (Figures 2 and 3).

A follow-up transthoracic echocardiogram five days after admission revealed improvement in left ventricular function, with a left ventricular ejection fraction off inotropes of 59%. Throughout the patient's hospital course, blood, urine, and sputum cultures remained negative.

## 3. Discussion

5-Fluorouracil (5-FU) is a pyrimidine analogue commonly used in the systemic treatment of gastrointestinal, genitourinary, and breast cancers. Capecitabine is an oral prodrug of 5-FU, which is enzymatically converted to active 5-FU in the tissues and which clinically resembles infusional 5-FU administration. Previous clinical studies estimate the rate of serious cardiac toxicity related to 5-FU at less than 2% [1]. Oral capecitabine carries a similar risk of cardiac toxicity [2]. Most of these events are related to coronary artery vasospasm, which can mimic signs and symptoms of acute coronary syndrome (ACS). This case describes acute cardiogenic shock related to infusional 5-FU, which is a far more serious and fortunately rare complication of this therapy.

In our case, severe coronary vasospasm may have resulted in acute left ventricular dysfunction leading to cardiogenic shock. Profound left ventricular dysfunction leading to shock following 5-FU therapy has rarely been reported, and therefore additional mechanisms of myocardial injury warrant consideration in this case. Such mechanisms could include direct myocardial injury, autoimmune-related endothelial damage, mitochondrial dysfunction, catecholamine surge analogous to Takotsubo cardiomyopathy, and abnormal accumulation of toxic metabolites [3, 4]. Previously published case reports have also implicated dihydropyrimidine dehydrogenase deficiency (DPD), a rare disorder which markedly increases toxicity related to 5-FU chemotherapy [5].

Hypersensitivity-associated myocarditis and acute hypersensitivity coronary syndrome (Kounis syndrome) have both been implicated as potential causes of coronary vasospasm related to medications, including antineoplastic agents such as 5-FU [6–8]. These conditions result from direct release of inflammatory mediators, such as leukotrienes and prostaglandins [9]. Leukotriene release from mast cell degranulation has been previously observed in patients treated with other chemotherapeutic agents, such as bleomycin and asparaginase [10]. In our case, eosinophil counts remained within normal ranges, and specific IgE levels or other markers of an acute allergic reaction were not measured. Ideally, IgEs, specific IgEs, histamine, neutral proteases, arachidonic acid derivatives, and specific cytokines should have been measured to investigate this possibility further [11].

In our patient's case, the rise in troponin, diffuse ST elevation on ECG, and echocardiographic findings were consistent with acute cardiomyopathy, mediated either by severe coronary spasm or through direct cardiac injury. ST elevation in leads II, III and aVF may also suggest a diagnosis of pericarditis. In most contexts, an ECG demonstrating ST elevation in contiguous leads would prompt consideration of coronary angiography. However, in the setting of recent 5-FU administration, this is sometimes deferred, as the most likely etiology is reversible coronary vasospasm. In our case, a coronary angiogram was never performed, and the complete reversal of wall motion abnormalities on subsequent echocardiogram is not in keeping with persistent coronary artery disease. Our patient is also unlikely to have significant DPD, as there was a lack of prolonged leukopenia, mucositis, or diarrhea.

Management of acute cardiogenic shock related to 5-FU is mainly supportive, with immediate discontinuation of the offending agent. Reversal of cardiomyopathy over several days is usually expected. After such severe cardiac toxicity, rechallenge with 5-FU should be considered contraindicated. If a hypersensitivity reaction is suspected, treatment with glucocorticoids and antihistamines would be reasonable, and an appropriate immunologic workup should be initiated [9, 11]. Medical prophylaxis with nitrates or calcium channel antagonists have never been proven to be effective in 5-FU induced coronary artery vasospasm [12, 13] and therefore have no established role in this setting, in spite of some case reports of successful prophylaxis [14]. Our patient went on to receive second-line chemotherapy consisting of gemcitabine and carboplatin, which was uneventful.

Cardiac complications related to chemotherapy are becoming an increasingly recognized cause of morbidity and mortality in cancer survivors. While cardiologists are aware of more common examples of chemotherapy-induced cardiac toxicity (such as anthracycline-related cardiomyopathy), it is important to maintain a high index of suspicion for less common presentations, such as 5-FU related coronary artery vasospasm and acute cardiac hypersensitivity.
